# Isolation of a Novel Peroxisomal Catalase Gene from Sugarcane, Which Is Responsive to Biotic and Abiotic Stresses

**DOI:** 10.1371/journal.pone.0084426

**Published:** 2014-01-02

**Authors:** Yachun Su, Jinlong Guo, Hui Ling, Shanshan Chen, Shanshan Wang, Liping Xu, Andrew C. Allan, Youxiong Que

**Affiliations:** 1 Key Laboratory of Sugarcane Biology and Genetic Breeding, Ministry of Agriculture, Fujian Agriculture and Forestry University, Fuzhou, Fujian, China; 2 The New Zealand Institute for Plant and Food Research, Sandringham, Auckland, New Zealand; Key Laboratory of Horticultural Plant Biology (MOE), China

## Abstract

Catalase is an iron porphyrin enzyme, which serves as an efficient scavenger of reactive oxygen species (ROS) to avoid oxidative damage. In sugarcane, the enzymatic activity of catalase in a variety (Yacheng05–179) resistant to the smut pathogen *Sporisorium scitamineum* was always higher than that of the susceptible variety (Liucheng03–182), suggesting that catalase activity may have a positive correlation with smut resistance in sugarcane. To understand the function of catalase at the molecular level, a cDNA sequence of *ScCAT1* (GenBank Accession No. KF664183), was isolated from sugarcane infected by *S. scitamineum*. *ScCAT1* was predicted to encode 492 amino acid residues, and its deduced amino acid sequence shared a high degree of homology with other plant catalases. Enhanced growth of ScCAT1 in recombinant *Escherichia coli* Rosetta cells under the stresses of CuCl_2_, CdCl_2_ and NaCl indicated its high tolerance. Q-PCR results showed that *ScCAT1* was expressed at relatively high levels in the bud, whereas expression was moderate in stem epidermis and stem pith. Different kinds of stresses, including *S. scitamineum* challenge, plant hormones (SA, MeJA and ABA) treatments, oxidative (H_2_O_2_) stress, heavy metal (CuCl_2_) and hyper-osmotic (PEG and NaCl) stresses, triggered a significant induction of *ScCAT1*. The ScCAT1 protein appeared to localize in plasma membrane and cytoplasm. Furthermore, histochemical assays using DAB and trypan blue staining, as well as conductivity measurement, indicated that *ScCAT1* may confer the sugarcane immunity. In conclusion, the positive response of *ScCAT1* to biotic and abiotic stresses suggests that *ScCAT1* is involved in protection of sugarcane against reactive oxidant-related environmental stimuli.

## Introduction

Sugarcane smut, a prevalent and worldwide disease of sugarcane, is caused by the basidiomycete *Sporisorium scitamineum* (*S. scitamineum*). The characteristic symptom of this disease is the emergence of black whips after three months of exposure to infection with smut [1]. The tainted buds may either produce symptoms, or exist as a latent infection and produce black whips in the following season [2]. The enormous quantity of teliospores as well as the quick spread within the sugarcane-producing area makes it almost impossible to completely eliminate this disease. Smut usually results in poor cane growth with profuse tillering, spindly shoots, and narrow leaves, therefore causing considerable loss in yield and sugar content [3]. The release of smut resistant sugarcane varieties, correct quarantine and integrated field management are three main pathways for the control this disease [1]. It is reported that the rates and patterns of colonization of *S. scitamineum* differ in resistant and susceptible sugarcane tissues [4]. Solas et al. found that buds of the resistant sugarcane cultivar were not subjected to intracellular penetration by *S. scitamineum* compared to that of the susceptible cultivar [5]. Susceptible cultivars produce a large number of sori which develop earlier than that in resistant cultivars [6]. Therefore, breeding for smut resistant sugarcane varieties has proved to be the most effective method [7].

Due to the complicated genetic background (a polyploid-aneuploid genome) and pressures of breeding selection (the interaction among sugarcane, smut pathogen and environmental factors), many years and multipoint resistance evaluation tests are needed to obtain relatively high smut resistant sugarcane variety [8]. Alternatively, genetic modification, with directional improvement and molecular assisted breeding technology linked to a target trait, is an alternative way to obtain a resistant variety more quickly and efficiently [9]. By introducing disease-resistance genes to improve gene expression, or by silencing disease-susceptible genes to increase resistance, genetic engineering has made it practical to generate smut resistant sugarcane cultivars [10].

Catalase (E.C.1.11.1.6; H_2_O_2_:H_2_O_2_ oxidoreductase; CAT) is an iron porphyrin enzyme, mostly localized in peroxisomes [11]. It serves as an efficient scavenger of reactive oxygen species (ROS). The main function of catalase is to remove excessive H_2_O_2_ (hydrogen peroxide) during developmental process or biotic/abiotic stress, to avoid oxidative damage [12]. Plant catalases are composed of a multi-gene family and have been reported in many plant species [13]. There are three members identified in *Arabidopsis thaliana* [14], *Nicotiana tabacum* and *Zea mays* [15], [16], two in *Hordeum vulgare* [17], one in *Solanum lycopersicum* [18]. In the catalase gene family, different members encode distinct catalase proteins that exhibit different patterns of subcellular localization and expression regulation [19].

The expression of various plant catalase genes is regulated temporally and spatially and responds to developmental and environmental oxidative stimuli [11], [13], [20], [21]. In *Panax ginseng, PgCat1* gene was expressed at different levels in leaves, stems, roots of *P. ginseng* seedlings and was induced by different stresses including heavy metals, osmotic agents, plant hormones and high light irradiances [11]. Kwon and An cloned a *Capsicum annuum* catalase cDNA, and northern hybridization showed its transcript was more abundant in stems than in leaves and roots, and more in the early stages than that in the mature stage of fruit development [19]. They also found that aluminum, sodium chloride (NaCl) and light treatment could induce its transcript. Previous research also revealed that the expression of three different maize catalase genes was regulated differentially in response to developmental phase or the fungal toxin cercosporin [22], abscisic acid (ABA) and salicylic acid (SA) [16], [22]. Wang et al. found increased transcription of a catalase gene (*MmeCAT*) in resistant clam *Meretrix meretrix* which indicated that *MmeCAT* could most probably benefit the immune system of clams to defend against pathogen infection [23]. The positive response of catalase genes to various stimuli suggested that catalase may help to protect the plant against reactive oxidant related environmental stresses. It is therefore interesting to determine the role of sugarcane catalases and their encoding genes in response to biotic and abiotic stresses.

To date, a partial cDNA sequence (GenBank Accession No. CF572408.1) similar to catalase has been cloned from *Saccharum* hybrid cultivar Q117 [24], while its function remained unclear. In the present research, we analyzed the differences of sugarcane catalase enzyme activity between Yacheng05–179 (resistant) and Liucheng03–182 (susceptible) inoculated with *S. scitamineum*. A novel full-length catalase gene *ScCAT1* (GenBank Accession No. KF664183) from sugarcane bud infected by *S. scitamineum* pathogen was cloned and characterized. Its response in *Escherichia coli* (*E. coli*) Rosetta strains, subcellular localization and expression patterns in sugarcane tissues under various biotic/abiotic stresses were reported. *Agrobacterium*-mediated transient expression of this gene in *Nicotiana benthamiana* (*N. benthamiana*) was used to functionally test ScCAT1.

## Materials and Methods

### Plant Materials and Treatments

Smut whips were collected in the most popular cultivar “ROC”22 in the Key Laboratory of Sugarcane Biology and Genetic Breeding, Ministry of Agriculture (Fuzhou, China), and stored at 4°C. Sugarcane varieties of Yacheng05–179 (smut resistant) and Liucheng03–182 (smut susceptible) were cultivated in the Key Laboratory of Sugarcane Biology and Genetic Breeding, Ministry of Agriculture (Fuzhou, China). All of the treatments were repeated independently three times.

For tissue distribution studies, six healthy 10 month old plants were selected. For each plant, the youngest fully expanded leaf viz +1 leaf with a visible dewlap (the collar between the leaf blade and sheath), all the buds, stem epidermis and the stem pith were taken for RNA extraction.

During biotic treatments, two-bud sets of both sugarcane genotypes, Yacheng05–179 and Liucheng03–182, were inoculated with 0.5 µL suspension containing 5×10^6^ spores·mL^−1^ in 0.01% (v/v) Tween-20, while controls were mock inoculated with 0.01% (v/v) Tween-20 in sterile distilled water instead of spores [25], [26]. All the inoculated sets were grown at 28°C in condition of 12 h light/12 h dark. Five buds from each of both genotypes were collected at each of the time point of 0 h, 6 h, 12 h, 24 h, 48 h, 72 h and 96 h. Samples were frozen in liquid nitrogen, and stored at −80°C.

During abiotic treatments, uniform four-month-old sugarcane tissue cultured plantlets of Yacheng05–179 were grown in water for one week and then transferred to the following seven different treatments in conical tubes at 28°C with 16 h light/8 h dark. The plantlets were treated with 5 mM SA solution, 25 µM MeJA (methyl jasmonate) in 0.1% (v/v) ethanol and 0.05% (v/v) Tween-20, 100 µM ABA, and 25% PEG (polyethylene glycol), and the plantlets were set to different periods of time (0 h, 6 h, 12 h and 24 h), respectively. In addition, plantlets were separately treated with 250 mM NaCl and 100 µM CuCl_2_ (copper chloride) for 0 h, 12 h, 24 h and 48 h [27], [28]_._ For H_2_O_2_ stress, the leaves were sprayed with 10 mM H_2_O_2_, and the sampling time points were 0 h, 6 h, 12 h and 24 h, respectively. After treatments, three sugarcane plantlets per time point were collected and immediately fixed in liquid nitrogen, and then kept at −80°C until used for analysis.

### Enzyme Extraction and Activity Assay

To analyze quantitative change in catalase activity in Yacheng05–179 and Liucheng03–182 after inoculation with smut pathogen, 0 h, 6 h, 12 h, 24 h, 48 h, 72 h and 96 h buds were sampled as above. Controls were mock inoculated with sterile distilled water. The frozen buds of 0.5 g were homogenized in a mortar and pestle with 3.0 mL of ice-cold phosphoric acid buffer (pH7.8) and a small amount of quartz sand. The supernatant was centrifuged at 4,000 ×*g* for 15 min at 4°C. The supernatant was used as a crude enzyme solution. After incubation at 25°C (for blank control, incubated in a boiling water for 10 min), 0.2 mL supernatant was mixed with 1.5 mL phosphoric acid buffer (pH7.8) added with polyvinylpyrrolidone (PVP) and 0.3 mL 0.1 mol/L H_2_O_2_ in 10 mL tube, which initiated the reaction. The decrease in absorbance was recorded by Lambda 35 UV WinLab software (Perkin Elmer, China) followed by the decomposition of H_2_O_2_ at 240 nm, and measured for a total of 3 min [29]. One unit of enzyme activity (U) was defined as A_240_ reduced 0.1 enzyme quantity per min per g. The enzyme activity was calculated as follows:
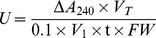


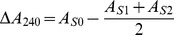



Among them, *A_S0_* means the absorbance of the blank control, *A_S1_* and *A_S2_* stand for the absorbance of the samples, *V_T_* means the total volume of the crude enzyme solution (mL), *V_1_* represents the volume of the detected crude enzyme solution (mL), *FW* means the fresh weight of sample (g) and t means the time from adding H_2_O_2_ to the last time (min). The activity of catalase was calculated by the activity level of inoculation minus the level of the mock at each corresponding time point.

### RNA Extraction

Total RNA of Yacheng05–179 and Liucheng03–182 was extracted with Trizol reagent (Invitrogen, China) according to the manufacturer’s protocol. The quality of the RNA was monitored by measuring the absorbance at 260 nm, 280 nm (NanoVue plus, GE, USA), and the 28 S and 18 S were examinated by electrophoresis. DNase I (Promega, China) was used to remove DNA contamination. The first-strand cDNA synthesis was completed by the Prime-Script™ RT Reagent Kit (TaKaLa, China).

### Isolation of Sugarcane Catalase Gene

Eighty-six sugarcane expressed sequence tags (ESTs), which share homology to the mRNA sequence of the sugarcane catalase gene (GenBank Accession No. CF572408.1), were obtained from sugarcane sequence database (cultivated sugarcanes (taxid:286192); wild sugarcane (taxid:62335); sugarcane (taxid:128810); sugarcane (taxid:4547)) in GenBank. The CAP3 sequence assembly program (http://pbil.univ-lyon1.fr/cap3.php) was used to construct a putative cDNA sequence of sugarcane catalase gene (*ScCAT1*). Then the cDNA of *ScCAT1* was amplified with primers designed on the basis of this assembled sequence.

Amplification of *ScCAT1* gene was performed with primers ScCAT1-cDNAF and ScCAT1-cDNAR (Table 1) on first-strand cDNA template of Yacheng05–179 post 48 h *S. scitamineum* inoculation in a Mastercycler (Eppendorf, Hamburg, Germany). The reaction was performed at 94°C for 5 min, and then subjected to 35 cycles of 94°C for 30 s, 57°C for 45 s and 72°C for 1 min, followed by a final extension at 72°C for 10 min. The expected length of the amplified fragments was 1,658 bp. PCR products were gel-purified, cloned into the pMD18-T vector (TaKaLa, China) and sequenced (Shenggong, China).

**Table 1:** Primers used in this study.

Primer	Sequence	Strategy
ScCAT1-cDNAF	GCGGCTTCCTACTCCTCGTCCTT	RT-PCR
ScCAT1-cDNAR	CGCCTGCTTTCTTCCTTGTCAATC	RT-PCR
ScCAT1-SublocF	TGCTCTAGAATGGATCCGTACAAGCAC	Subcellular localization vector construction
ScCAT1-SublocR	GGACTAGTCATGTTTGGCTTCAGGTTCAG	Subcellular localization vector construction
ScCAT1-32aF	CGGAATTCATGGATCCGTACAAGCAC	prokaryotic expression vector construction
ScCAT1-32aR	CCCTCGAGTTACATGTTTGGCTTCAGGT	prokaryotic expression vector construction
GAPDH-QF	CACGGCCACTGGAAGCA	Q-PCR
GAPDH-QR	TCCTCAGGGTTCCTGATGCC	Q-PCR
ScCAT1-QF	CTCTGCTCCTCCAATCCC	Q-PCR
ScCAT1-QR	GAGTGACCTCAAAGAAACCCT	Q-PCR
ScCAT1-1301F	GCTCTAGAATGGATCCGTACAAGCACCG	Over expression vector construction
ScCAT1-1301R	TCCCCCGGGTTACATGTTTGGCTTCA	Over expression vector construction

### Protein Structural Analysis and Phylogenetic Tree Construction

Sequence data were analyzed by ORF (open reading frame) Finder (http://www.ncbi.nlm.nih.gov/gorf/gorf.html), ProtParam (http://web.expasy.org/protparam/), SignalP 4.0 Server (http://www.cbs.dtu.dk/services/SignalP/), TargetP 1.1 server (http://www.cbs.dtu.dk/services/TargetP/), SMART (http://smart.embl-heidelberg.de/), PSORT Prediction (http://psort.hgc.jp/form.html). After blast comparison, the amino acid sequence of *ScCAT1* was aligned with published plant catalases, including *Sorghum bicolor* catalase (XP_002437631.1), *Z. mays* catalase (NP_001241808.1), *Oryza sativa* catalase (A2YH64.2), *Brachypodium distachyon* catalase (XP_003563243.1), *Puccinellia chinampoensis* catalase (ADN94253.1), *H. vulgare* catalase (P55307.1), *Triticum aestivum* catalase (P55313.1) and *Setaria italica* catalase (XP_004966515.1). Multiple alignment of the amino acid sequences was carried out using the Clustal W software. The phylogenetic tree was constructed following the neighbor-joining (NJ) method (1,000 bootstrap replicates) by using the MEGA 5.05 software [13].

### *Agrobacterium*-mediated Transient Expression and Subcellular Localization Assay

For the studying of subcellular location constructs of pCAMBIA 2300-GFP were generated, *ScCAT1* gene was PCR amplified from pMD18-T-*ScCAT1* using primers ScCAT1-SublocF and ScCAT1-SublocR (*Xba* I and *Spe* I sites) as indicated in Table 1. The fragment was inserted into the vector of pCAMBIA 2300-GFP to construct the fusion protein expression vector of *35S*::*ScGluA1*::*GFP* (Fig. S1). The recombinant plasmid, was verified by PCR, double digestion and sequencing followed by transfection of the competent cells of *Agrobacterium tumefaciens* strain EHA105.

The assay for *Agrobacterium*-mediated transformation referred to the method as previously described [30]. *Agrobacterium* strain EHA105 carrying the indicated construct was grown overnight in LB liquid medium containing 35 µg·mL^−1^ rifampicin and 50 µg·mL^−1^ kanamycin. The suspension at OD_600_ = 0.8 (containing 200 µM acetosyringone) was infiltrated into 4–5 weeks old *N. benthamiana* leaves and cultured at 24°C for 2 days (16 h light/8 h darkness). The subcellular localization of the fusion protein was visualized using fluorescence microscopy (Axio Scope A1, Germany).

### Expression in *Escherichia coli* Rosetta Cells

To study the function of *ScCAT1* in prokaryotes, the *ScCAT1* ORF was amplified by PCR from the identified cDNA clone using the primers ScCAT1-32aF and ScCAT1-32aR (Table 1) followed by 94°C for 4 min; 94°C for 30 s, 58°C for 30 s, 72°C for 1.5 min, 35 cycles; and 72°C for 10 min. The *ScCAT1* ORF with *Eco*R I and *Xho* I sites was subcloned into pET 32a (+) vector with *Eco*RI-*Xho*I sites in the *E. coli* Rosetta strains to generate the putative recombinant (pET 32a-*ScCAT1*). The desired recombinant plasmid was identified by PCR amplification, double digestion and sequencing. The prokaryotic expression product was induced in 1.0 mM isopropyl β-D-thiogalactoside (IPTG) for 8 h at 37°C and analyzed by sodium dodecyl sulfate-polyacrylamide gel electrophoresis (SDS-PAGE). Meanwhile, LB medium with blank *E. coli* Rosetta strains (blank) or Rosetta+pET 32a (control) was each induced in IPTG for 8 h and also analyzed by SDS-PAGE [26], [31].

During the response of *E. coli* cells to various abiotic stresses, the growth of *E. coli* Rosetta strains transformed with pET 32a and pET 32a-*ScCAT1* was analyzed using spot assay in different treatments of CuCl_2_, CdCl_2_ or NaCl. When OD_600_ of the LB medium (plus 170 µg·mL^−1^ chloramphenicol and 80 µg·mL^−1^ ampicillin) with *E. coli* cells reached 0.6, IPTG was added to a final concentration of 1.0 mM, and then continued growth for 12 h at 37°C. Thereafter, the cultures were diluted to 0.6 (OD_600_), and then to two levels (10^−3^ and 10^−4^). Ten microlitres from each dilutions was spotted on LB plates (plus 170 µg·mL^−1^ chloramphenicol and 80 µg·mL^−1^ ampicillin) containing CuCl_2_ (250, 500 and 750 µM), CdCl_2_ (250, 500 and 750 µM) or NaCl (250, 500 and 750 mM) [26], [31]. All these plates were cultured in 37°C overnight and photographed.

The effect of 750 µM CuCl_2_, 750 µM CdCl_2_ and 250 mM NaCl on the growth of *E. coli* strains with pET 32a-*ScCAT1* or pET 32a was studied in LB medium followed with Su et al. [26]. As above, when cells were grown as earlier described and then diluted to 0.6 (OD_600_), 400 µL of cells were transferred into 10 mL of LB medium containing 170 µg·mL^−1^ chloramphenicol and 80 µg·mL^−1^ ampicillin, 750 µM CuCl_2_ or 750 µM CdCl_2_ or 250 mM NaCl [32]. Cultures were shaken at 200 rpm at 37°C and growth of the cells was measured at every 2 h by Lambda 35 UV WinLab software (Perkin Elmer, USA).

### Real-time Quantitative PCR Analysis

The each time point of 0 h, 6 h, 12 h, 48 h and 72 h during Yacheng05–179-smut incompatible interaction and Liucheng03–182-smut compatible interaction, as well as mock plants inoculated with sterile distilled water at each corresponding time point, were used to analyze the expression patterns of the *ScCAT1*. The relative expression of the target gene under certain biotic stress was calculated by the expression level of the inoculated sample minus the level of the mock at each corresponding time point. For tissue-specific expression of *ScCAT1*, the leaf, bud, stem epidermis and stem pith of sugarcane variety Yacheng05–179 were used as experimental materials. The expression of *ScCAT1* under the stresses of SA, MeJA, ABA, H_2_O_2,_ PEG, CuCl_2_ and NaCl were also performed by real-time quantitative PCR (Q-PCR).

The method of Q-PCR followed the instruction of the SYBR Green Master (ROX) (Roche, China) on a 7500 Q-PCR system (Applied Biosystems, USA). The *GAPDH* gene (GAPDH-QF/GAPDH-QR) (Table 1) was chosen as the internal control of the Q-PCR [28]. According to the sequence of *ScCAT1*, a pair of specific primers ScCAT1QF/ScCAT1-QR was designed using the Primer Premier 5.0 software. Q-PCR was carried out with FastStart Universal SYBR Green Master (ROX) in a 20 µL volume containing 10 µL FastStart Universal SYBR Green PCR Master (ROX), 0.5 µM of each primer and 2.0 µL template (100 × diluted cDNA). PCR with distilled water as template was performed as control. The Q-PCR reaction condition was held at 50°C for 2 min, 95°C for 10 min, 40 cycles of 95°C for 15 s and 60°C for 1 min. When the reaction was complete, the melting curve was analyzed. Each Q-PCR was repeated three times. The 2^−△△Ct^ method was adopted to analyze the Q-PCR results [33].

### Histochemical Assay

For analysis of defense response caused by *ScCAT1* over-expression, primers of ScCAT1-1301F/ScCAT1-1301R in Table 1 (*Xba* I -*Sma* I sites) were used to construct binary vector expressing *ScCAT1* (pCAMBIA 1301-*ScCAT1*). *Agrobacterium* strain EHA105 containing recombinant vector and pCAMBIA 1301 vector alone were grown overnight in LB liquid medium (plus 35 µg·mL^−1^ rifampicin and 50 µg·mL^−1^ kanamycin) at 28°C. Then cultures were pelleted and resuspended in MS liquid medium (plus 200 µM acetosyringone) at OD_600_ = 0.8 and infiltrated into *N. benthamiana* leaves at eight-leaf stage [34]. Plants were incubated at 24°C for 1–2 days (16 h light/8 h darkness), which were employed to following different tests.

DAB (3,3′-diaminobenzidinesolution) staining. Agroinfiltrated leaves were incubated in 1.0 mg·mL^−1^ DAB-HCl solution in the dark overnight. Then the leaves were destained by boiling in 95% ethanol for 5 min. The bronzing color of the leaves for H_2_O_2_ detection which generated in leaves after treatments was photographed [35].

Trypan blue staining. The infiltrated leaves were boiled for 5 min in lactophenol-ethanol trypan blue solution (10 mL glycerol, 10 mL lactic acid, 10 g phenol, 10 mg trypan blue, 30 mL absolute ethanol and 10 mL distilled water). Then the leaves were destained in 2.5 g·mL^−1^ choloral hydrate in distilled water and the blue color indicates the cell death [30].

Measurement of ion conductivity. It was performed as previously described with some modifications [36]. Six leaf discs (11 mm in diameter per leaf) were cut and washed in distilled water and then incubated in 20 mL of distilled water and shaken slowly at room temperature for 60 min. After that, electrolyte leakage was measured using a conductivity meter (SevenEasy, METTLER TOLEDO, Switzerland).

## Results

### Enzyme Activity of Catalase

**Figure 1:**
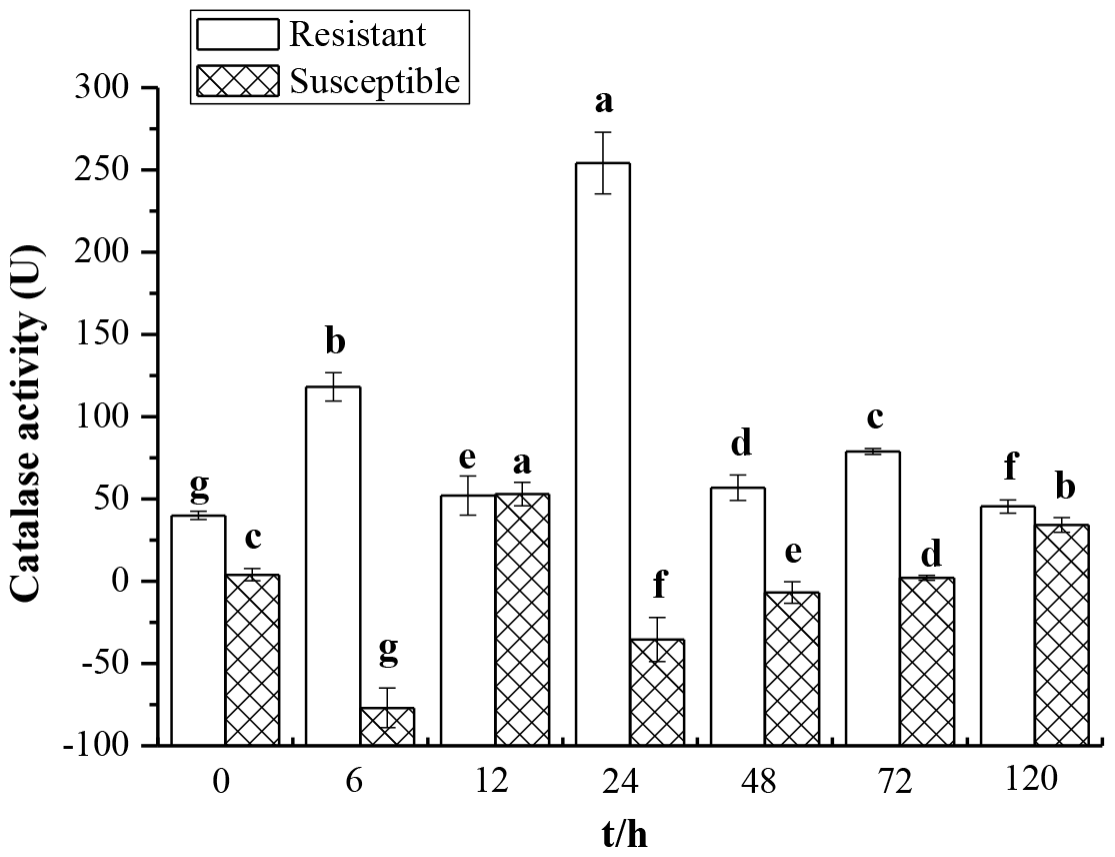
The catalase activity in smut resistant (Yacheng05–179) and smut susceptible (Liucheng03–182) sugarcane varieties inoculated with *Sporisorium scitamineum*. All data points (deduction its mock) are means±SE (n = 3).

To analyze the correlation between catalase activity and smut resistance, the changes in enzyme activity in smut challenged Yacheng05–179 (resistant) and Liucheng03–182 (susceptible) cultivars were studied and different patterns of enzyme activity change were found. As shown in Fig. 1, activity of catalase in Yacheng05–179 increased at 6 h (118.19 U) and reached the peak value of 254.14 U at 24 h compared to its mock. It should be noted that the catalase activity in the resistant sugarcane variety (Yacheng05–179) was always higher than that of the mock at all the sampling time points, but the tendency was an increased at 12 h and 120 h, decreased at 6 h and 24 h and almost unchanged at 0 h, 48 h, 72 h in the susceptible one (Liucheng03–182). In addition, when compared with the mock, the catalase activity was much higher in the resistant variety (from 40.00 U to 254.14 U) than that in the susceptible one (from −76.94 U to 52.97 U) at all the time points. These results suggest that there are positive correlations between catalase activity and smut resistance in sugarcane.

### Cloning and Sequence Analysis of *ScCAT1*

To study the sugarcane catalase at the molecular level, a 1,658 bp full-length catalase gene *ScCAT1* (GenBank Accession No. KF664183) was cloned using RT-PCR method combined with *in silico* cloning technique. There were 1,479 nucleotides in its ORF (Fig. S2). *ScCAT1* encoded a predicted polypeptide of 492 amino acids with no signal peptide. The predicted protein had a molecular mass of 56.81 kDa with a pI of 6.72. A search at the NCBI for conserved protein domains indicated that ScCAT1 belonged to a member of catalase-like superfamily. The catalytic active site and heme binding motifs of ScCAT1 were detected by Motif Scan Online program. 17 amino acids at the position of 54–70 (FDRERIPERVVHARGAS) were reported to be a catalase active site signature, and the heme-ligand signature was detected at the position of 344–352 (RIFSYADTQ). These data suggested clearly that sugarcane *ScCAT1* encoded a putative peroxisomal catalase. Furthermore, it also predicted that ScCAT1 contains no transmembrane helix domain, implying that ScCAT1 is not a membrane located or secretory protein.

**Figure 2:**
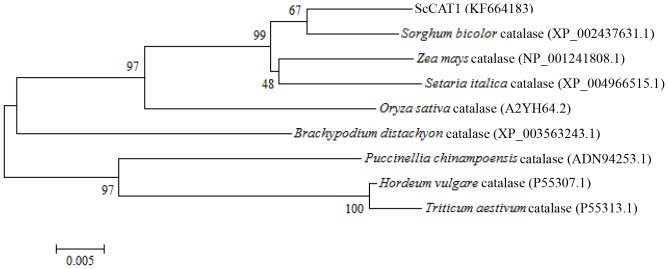
Phylogenetic trees based on catalase amino acid sequences, showing the phylogenetic relationships between ScCAT1 (KF664183) and the catalases from other plant species. Neighbor-joining method was used.

A GenBank Blastp comparison showed that ScCAT1 exhibited high identity with other plant catalases, including *S. bicolor* catalase (XP_002437631.1) (97.97% identity), *Z. mays* catalase (NP_001241808.1) (97.56% identity), *O. sativa* catalase (A2YH64.2) (94.92% identity), *B. distachyon* catalase (XP_003563243.1) (93.29% identity), *P. chinampoensis* catalase (ADN94253.1) (92.07% identity), *H. vulgare* catalase (P55307.1) (91.67% identity), *T. aestivum* catalase (P55313.1) (91.26% identity) and *S. italica* catalase (XP_004966515.1) (87.02% identity). Phylogenetic analysis (Fig. 2) revealed that ScCAT1 was closely related to *S. bicolor* catalase (XP_002437631.1), Z. *mays* catalase (NP_001241808.1) and *S. italica* catalase (XP_004966515.1), with the homologies of 97.97%, 97.56% and 87.02%, respectively.

### Subcellular Localization of ScCAT1

**Figure 3:**
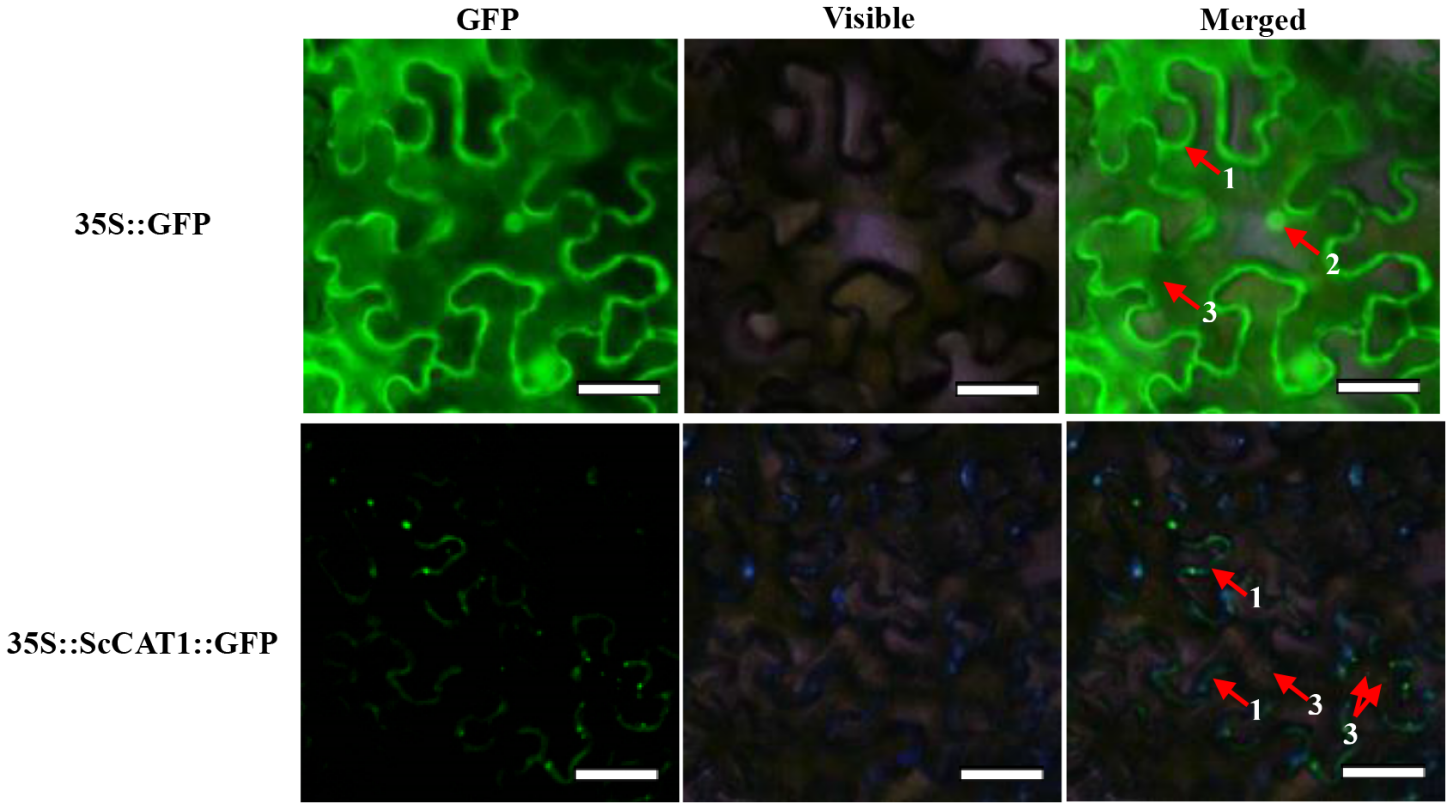
Subcellular localizations of ScCAT1 and empty vector in *Nicotiana benthamiana* leaves 48 h after infiltration. The epidermal cells were used for taking images of green fluorescence, visible light and merged light. Read arrows 1, 2 and 3 indicated plasma membrane, nucleus and cytoplasm, respectively. Bar = 50 µm.

To further understand the function of *ScCAT1* gene, its subcellular localization was conducted. *ScCAT1* was recombined into plant expression vector pCAMBIA 2300 between the sites of the *35S* promoter and *GFP* (Figs. S1 and S3), and its location was characterized by transient expression of the target gene and GFP in *N. benthamiana* leaves with *Agrobacterium*-mediated transformation. After 2 days of cultivation, the infiltrated leaves were harvested and the reporter protein GFP was observed under a fluorescence microscope. The results revealed that 35S::ScCAT1::GFP was located in plasma membrane and cytoplasm (Fig. 3). In contrast, GFP was shown in the nucleus, cytoplasm and plasma membrane cells transiently transfected with 35S::GFP.

### Expressions of ScCAT1 in *E. coli*

As reported before, different stresses, such as copper (Cu), cadmium (Cd), high temperature, wounding, ethylene (ET), H_2_O_2_, SA, jasmonic acid (JA), ABA and other inducers, could trigger an induction of plant catalases [11], [13], [21], [37], [38]. To study the function of ScCAT1 in response to different kinds of adverse environments *in vivo*, pET 32a-*ScCAT1* (Fig. S4A) was transformed into *E. coli* Rosetta cell. The recombinant protein of 62 kDa was specifically induced and accumulated approximately after 8 h IPTG induction on the SDS-PAGE (Fig. S4B).

**Figure 4:**
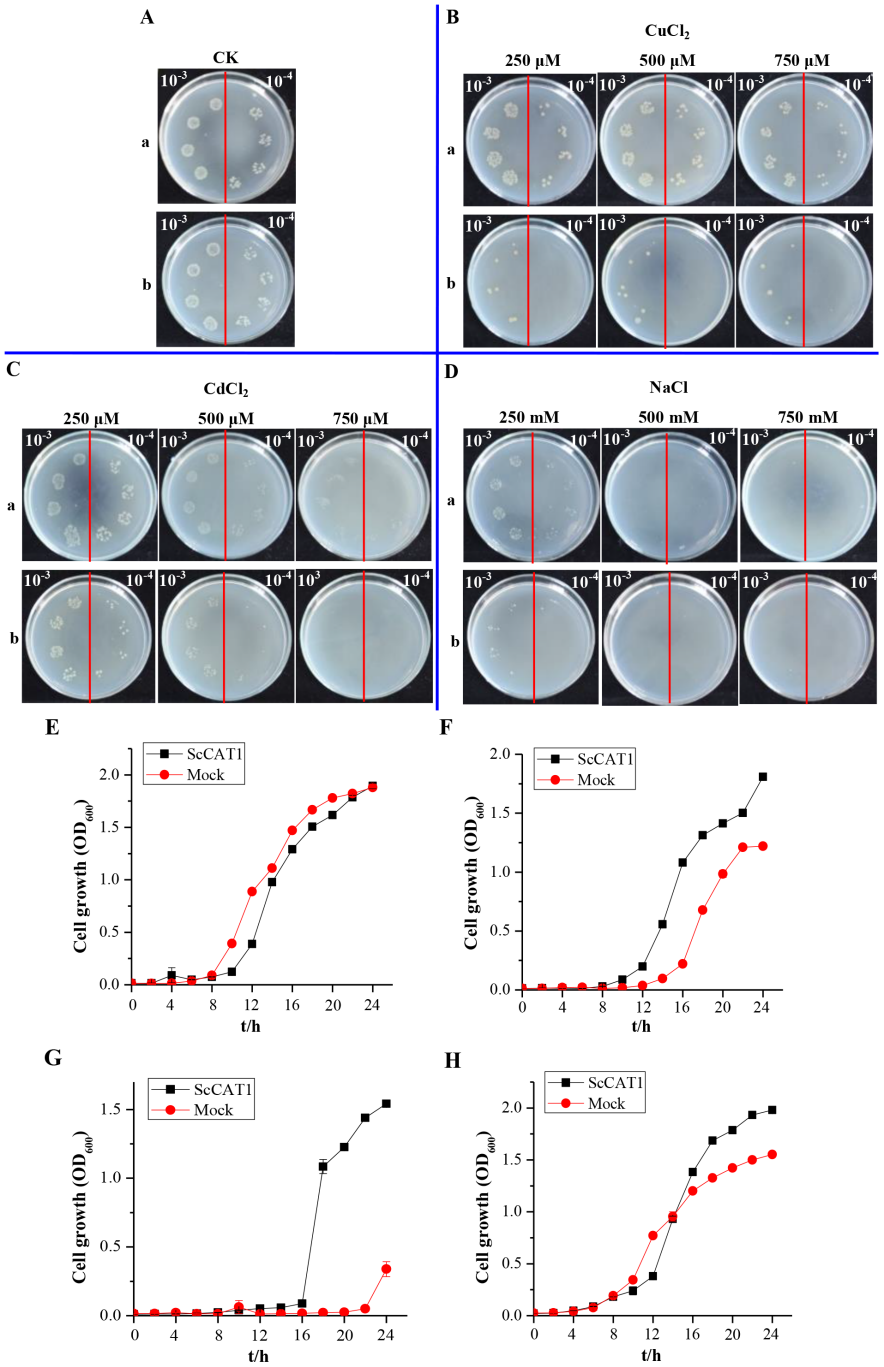
Spot assays of Rosetta+pET 32a-*ScCAT1* (a) and Rosetta+pET 32a (mock) (b) on LB plates with CuCl_2_, CdCl_2_ and NaCl (A–D). And liquid culture assay in LB liquid medium with 750 µM CuCl_2_, 750 µM CdCl_2_ and 250 mM NaCl (E–H). IPTG (isopropyl β-D-thiogalactoside) was added to the cultures of Rosetta+pET 32a-*ScCAT1* and Rosetta+pET 32a to induce the expression of recombinant protein. The cultures were adjusted to OD_600_ = 0.6. Ten microliters from 10^−3^ (left side of red line on plate) to 10^−4^ (right side of red line on plate) dilutions were spotted onto LB basal (A) plates or with CuCl_2_ (250, 500 and 750 µM) (B), CdCl_2_ (250, 500 and 750 µM) (C), NaCl (250, 500 and 750 mM) (D). For studying the growth analysis of *ScCAT1*, Rosetta+pET 32a-*ScCAT1* and Rosetta+pET 32a were grown in LB liquid medium with LB basal medium (E) or with 750 µM CuCl_2_ (F), 750 µM CdCl_2_ (G), and 250 mM NaCl (H). All data points are means±SE (n = 3). CuCl_2:_ copper chloride; CdCl_2_: cadmium chloride; NaCl: sodium chloride.

The growth of gene-expressed cells (Rosetta+pET 32a-*ScCAT1*) and mock (Rosetta+pET 32a) was analyzed on LB plates with different supplements (Figs. 4A, B, C, and D). After one day culture, Rosetta+pET 32a-*ScCAT1* showed an increased number of colonies as compared to the control cells on LB plates containing CuCl_2_, CdCl_2_ and NaCl. The growth was also analyzed in the LB liquid medium containing 750 µM CuCl_2_, 750 µM CdCl_2_ and 250 mM NaCl (Figs. 4E, F, G and H). All the Rosetta+pET 32a-*ScCAT1* cells showed faster growth as compared to that of the mock which revealed that ScCAT1 had an effect on increasing the tolerance to CuCl_2_, CdCl_2_ and NaCl. These results demonstrat that the recombinant protein of ScCAT1 enhanced growth ability of prokaryotic *E. coli* Rosetta strains in stress conditions.

### Tissue-specific Expression Analysis of *ScCAT1*

**Figure 5:**
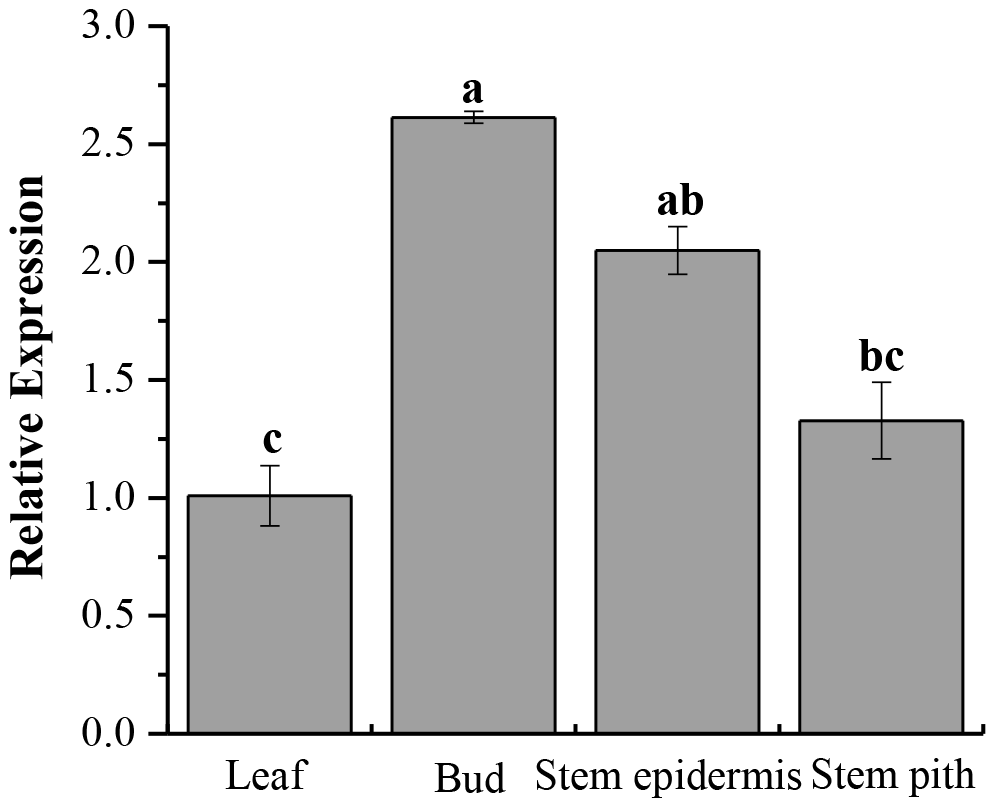
Tissue-specific expression analysis of the *ScCAT1* in sugarcane variety Yacheng05–179. Data are normalized to the *GAPDH* expression level. All data points are means±SE (n = 3).

The relative expression of *ScCAT1* was detected in four kinds of sugarcane tissues, including leaf, bud, stem epidermis and stem pith. As showed in Fig. 5, the bud exhibited the highest mRNA expression, while the mRNA expression of stem epidermis and stem pith was at a moderate level. The leaf showed a relatively low level in comparison with the other three kinds of tissues.

### *ScCAT1* Expression in Response to Different Stress Treatments

**Figure 6:**
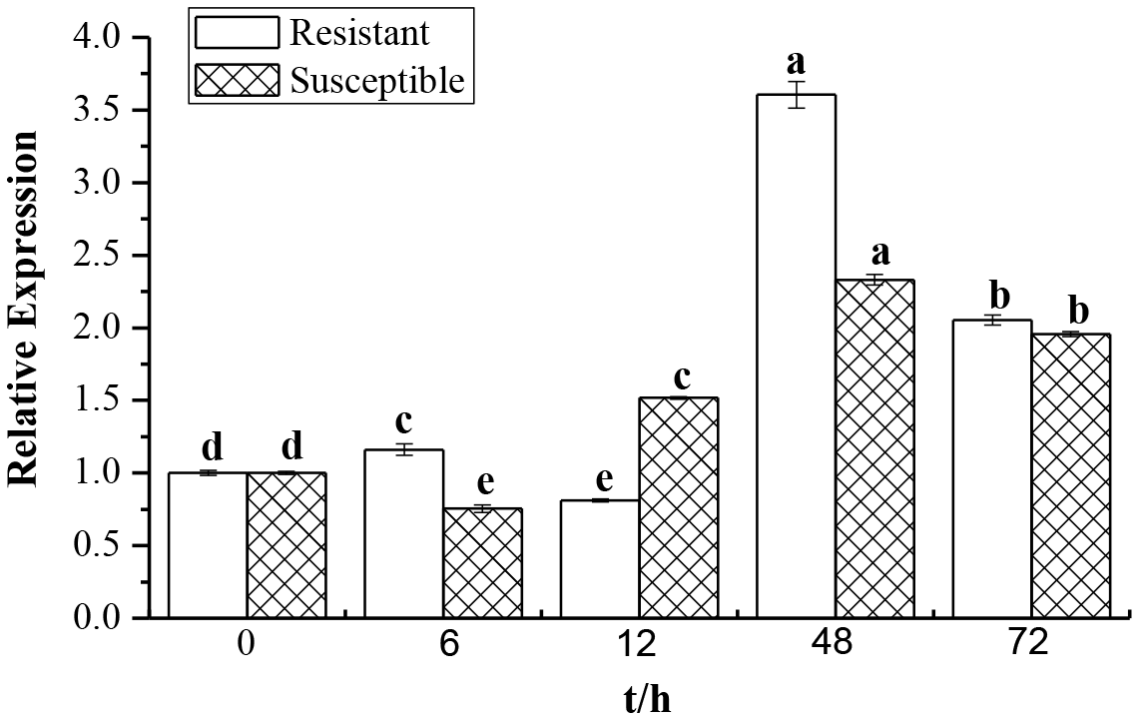
Q-PCR analysis of the *ScCAT1* expression patterns in biosystem of sugarcane-smut (*Sporisorium scitamineum*) interaction. Data was normalized to the *GAPDH* expression level. All data points (deduction its mock) are means±SE (n = 3). Resistant: Yacheng05–179 variety; Susceptible: Liucheng03–182 variety.

Smut challenged sugarcane (Yacheng05–179 and Liucheng03–182) buds were detected by Q-PCR for examination whether the expression of *ScCAT1* was induced or inhibited (Fig. 6). In order to eliminate the influence of wounding, the relative expression of the target gene was calculated by the expression level of the inoculated sample minus the level of the mock at each corresponding time point. As indicated in Fig. 6, after the inoculation of smut pathogen, the mRNA expression of *ScCAT1* in resistant variety Yacheng05–179 was higher than that in susceptible variety Liucheng03–182. During the sugarcane-smut incompatible interaction, the transcript of *ScCAT1* in Yacheng05–179 began was elevated as early as 6 h post-inoculation (6 hpi), while that of *ScCAT1* in Liucheng03–182 appeared delayed (12 hpi). Furthermore, the transcript of *ScCAT1* in Yacheng05–179 and Liucheng03–182 reached the maximum at 48 h, but the expression in incompatible interaction was 1.55 times that of the compatible one, and then decreased in both. During the whole process of interaction, the transcript of *ScCAT1* in the incompatible cultivar almost always higher than that of the compatible, except at 12 h. These data reveal that the up-regulation of *ScCAT1* expression was most probably associated with smut resistance in sugarcane.

**Figure 7:**
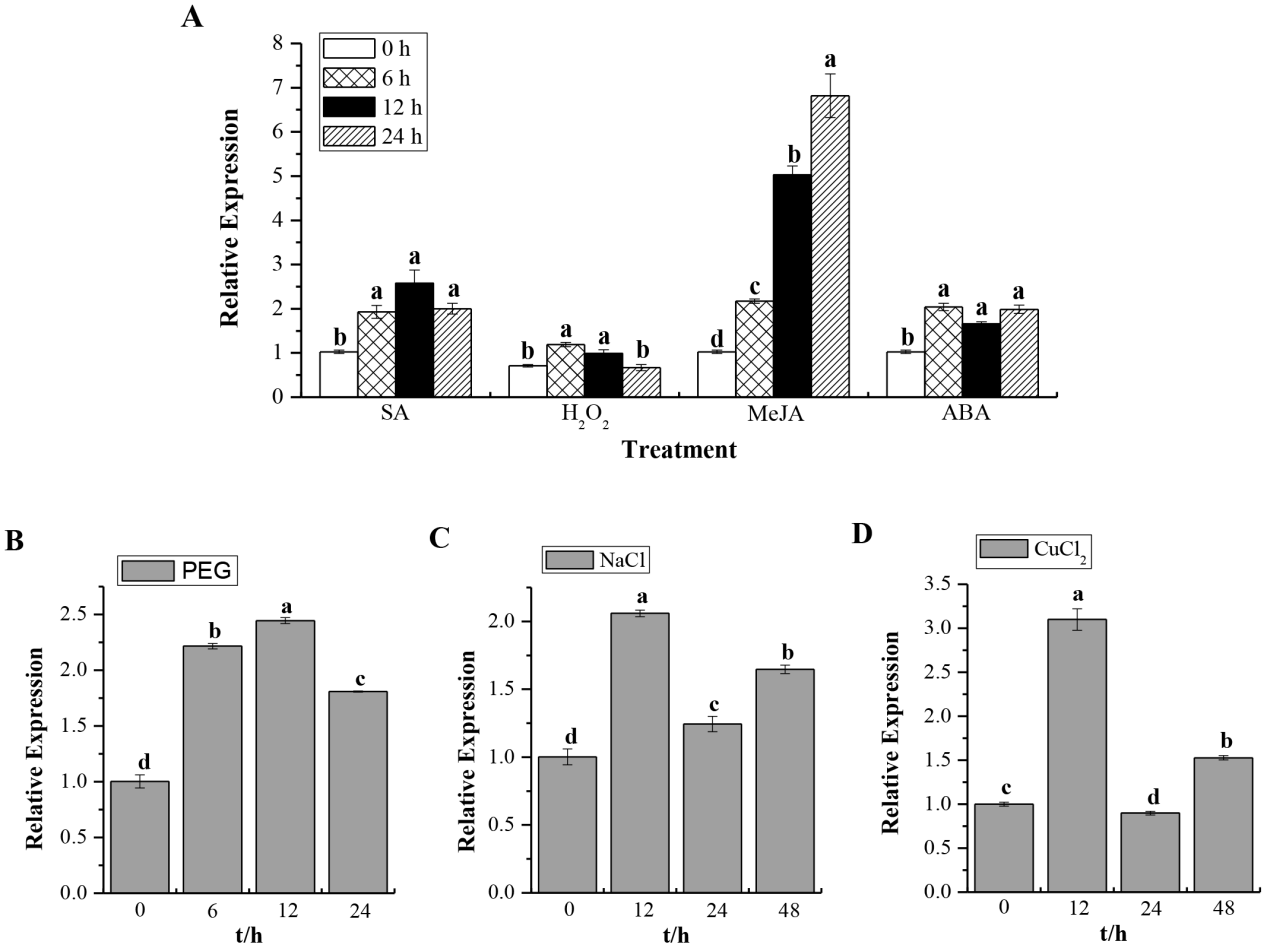
Q-PCR analysis of the *ScCAT1* expression patterns in Yacheng05–179 plantlets with abiotic elicitors. Data are normalized to the *GAPDH* expression level. (**A**) The relative expression of *ScCAT1* under the stresses of 5 mM SA, 10 mM H_2_O_2_, 25 µM MeJA and 100 µM ABA. (**B**) The relative expression of *ScCAT1* under 25% PEG stress. (**C**) The relative expression of *ScCAT1* under 250 mM NaCl stress. (**D**) The relative expression of *ScCAT1* under 100 µM CuCl_2_ stress. All data points are means±SE (n = 3). SA: salicylic acid; H_2_O_2_: hydrogen peroxide; MeJA: methyl jasmonate; ABA: abscisic acid; PEG: polyethylene glycol; NaCl: sodium chloride; CuCl_2:_ copper chloride.

Expression of *ScCAT1* in response to various abiotic stimuli in Yacheng05–179 plantlets was checked after treatment with 5 mM SA, 10 mM H_2_O_2_, 25 µM MeJA, 100 µM ABA, 25% PEG, 250 mM NaCl and 100 µM CuCl_2_, and the results shown in Fig. 7. Interestingly, *ScCAT1* showed a positive response to exogenous stresses, including plant hormones stresses of SA, MeJA and ABA, oxidative stress of H_2_O_2_, hyper-osmotic stresses of PEG and NaCl, as well as mental stress of CuCl_2_. *ScCAT1* transcription was always up-regulated and the expression level usually increased steadily from 0 h to 24 h or 48 h after post-treatment with these seven exogenous inducers. These results suggest that *ScCAT1* may be a positive responsive component of abiotic stress in sugarcane.

### Transient Over-expression of *ScCAT1* in *N. benthamiana* Leaves Induces Hypersensitive Reaction Response

**Figure 8:**
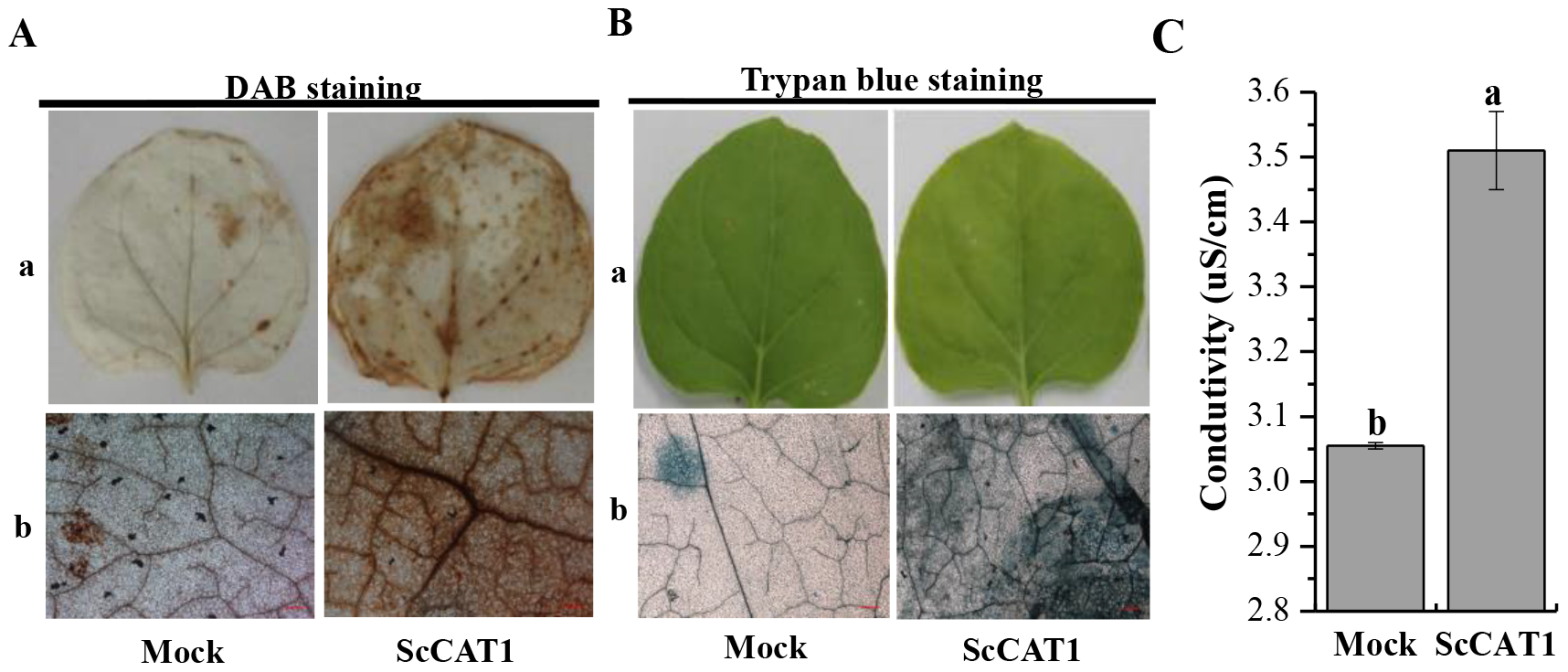
The effect of transient over-expression of *ScCAT1* on immunity induction in *Nicotiana Benthamiana* leaves. (**A**) DAB staining with *N. benthamiana* leaves 48 h after *35S::ScCAT1*-containing *Agrobacterium* strain infiltration to assess the H_2_O_2_ production; a: images taken by SONY camera; b: images taken by microscope. (**B**) Cell death measured by trypan blue staining of transient expression leaves 5 d after agroinfiltration; a: phenotypes of *N. Benthamiana* 5 d after infiltration taken by SONY camera; b: images of trypan blue staining taken by microscope. (**C**) Conductivity measurement of *N. Benthamiana* leaves infiltrated with *35S::ScCAT1*-containing *Agrobacterium* strain after 48 h. Mock: *Agrobacterium* strain carrying *35S::00*. Bar = 10 µm. All data points are means±SE (n = 3).

To test whether *ScCAT1* can induce HR and immunity in plant, *ScCAT1* was transient over-expressed in *N. benthamiana* leaves by infiltration with *Agrobacterium* EHA105 carrying pCAMBIA 1301 (mock) and pCAMBIA 1301-*ScCAT1*. The results showed that at the time point of 48 h after infiltration, a typical HR symptom, darker DAB staining and enhanced electrolyte leakage, was found in the leaves expressing the target gene (Figs. 8A and C). Furthermore, injected leaves 5 d after agroinfiltrated by *35S::ScCAT1* presented yellow symptoms (Fig. 8B). What is more, cell death measured by qualitative trypan blue staining showed a darker color than that in mock (Fig. 8B). These results indicate the involvement of *ScCAT1* in cell death responses.

## Discussion

Fungal disease is a major concern worldwide for sugarcane production and most other crops. During plant-pathogen interactions, many antifungal components have been identified [39]. Peroxidase (POD) activity increased in resistant sugarcane varieties (and not in susceptible) implies that it may be related to smut resistance [40]. Our previous report showed that β-1,3-glucanase activity in the resistant variety increased faster and lasted longer than that of the susceptible one after challenged by *S. scitamineum*, which showed a positive relationship between the activity of the sugarcane β-1,3-glucanase and smut resistance [26]. Plant catalase, one of the scavenger enzymes, has been also shown to be involved in plant defense and development [41]. Wang et al. found that catalase could be induced by pathogen infection in resistant clam *Meretrix meretrix* [23]. As showed in Fig. 1, the activity of catalase increased in the resistant genotype (Yacheng05–179) after challenged by *S. scitamineum* in comparison to the susceptible cultivar (Liucheng03–182). There appears to be a positive correlation between catalase activity and sugarcane smut resistance. This observation should be repeated in different resistant genotypes.

The capacity of a plant to scavenge H_2_O_2_ may result from increased activities of scavenger enzymes or up-regulated expression of genes increasing of the levels of the corresponding proteins [42]. Multiple catalase isozymes in plants have been observed. Previous research demonstrated that there were at least six catalase isozymes existing in *A. thaliana* encoded by a multi-gene family including three genes (*cat1*, *cat2* and *cat3*) [14]. In *Z. mays*, three catalase isoenzymes encoded by three different structural genes were observed [38]. A sweet potato catalase *SPCAT1* was cloned from mature leaves treated with ethephon and found that it could alleviate ethephon-mediated leaf senescence and H_2_O_2_ elevation [13]. Until now, there has been no report on sugarcane catalase genes involved in the sugarcane-smut interaction. In this study, we isolated and characterized a full-length sugarcane catalase gene *ScCAT1* which encoded a polypeptide of 492 amino acids and had high identities with several other plant catalases. Using the method of *Agrobacterium*-mediated transformation in *N. benthamiana* leaves, 35S::ScCAT1::GFP was located in plasma membrane and cytoplasm in cells (Fig. 3) which is consistent with a previous report that catalase mostly localized in peroxisomes, glyoxysome and cytoplasm [11], [43].

Recent publications have reported that *E. coli* cells can be enhanced or inhibited under stress expressing recombinant proteins [27], [31], [32], [44]. Some of the protective mechanisms were similar in both eukaryotes and prokaryotes under stress stimuli [45]. Gupta et al. studied an A-2 type DREB transcription factor from extreme halophyte *Salicornia* brachiata and found it conferred abiotic stress tolerance in *E. coli* cells under NaCl, PEG and mannitol treatments, which may be due to the stress regulated function by this transcription factor [32]. Guo et al. tested a sugarcane dirigent protein gene *ScDir* and a metallothionein gene *ScMT2-1-3* in the *E. coli* system, which indicated that they offered different tolerance against PEG, NaCl and mental stresses [27], [31]. Chaurasia et al. studied that phytochelatin synthase gene *PCS,* when expressed in *E. coli*, provided better protection against the stresses of heat, salt, carbofuron, cadmium, copper and UV [44]. In the present study, the ScCAT1 recombinant protein expressed in *E. coli* Rosetta cells leads to a better growth under the stresses CuCl_2_, CdCl_2_ and NaCl. In eukaryote, the previous studies found that the increased tolerance to stress maybe due to the activity and expression of scavenging enzymes which increased in plants placed in different conditions [12]. It has been proposed that catalase, one of the antioxidant enzymes, can be modulated and controlled in response to excessive iron stress, due to alterations in the electron transport chain and damages to the thylakoidal membranes [46]. Therefore, it is plausible to predict that the ScCAT1 encoded by *ScCAT1* gene cloned in this study could be helpful for the tolerance/stresses of sugarcane to CuCl_2_, CdCl_2_ and NaCl.

The plant faces variable environmental stresses like soil salinity, temperature, drought and cold, and may often present a series of physiological and biochemical changes which are a highly complex and disturb plant growth and yield. To examine the accumulation of sugarcane catalase gene in different developmental processes and environmental conditions, the expression of *ScCAT1* gene in sugarcane was analyzed by Q-PCR method (Figs. 5, 6 and 7). Results indicated that while expressed at moderate levels in stem epidermis and stem pith, *ScCAT1* was expressed at a relatively high level in the bud (Fig. 5). Similar to other species of *Ustilago*, the *S. scitamineum* is a parasite of young meristematic tissues and gains entry into the host, exclusively through the bud scales [47]. From above, high expression of *ScCAT1* in sugarcane bud may help to defend against the smut pathogen. In our study, the target transcript of *ScCAT1* was found to be higher in the incompatible interaction than that in the compatible one during sugarcane-*S. scitamineum* interaction (Fig. 6). After the smut pathogen challenge in Yacheng05–179, the expression of *ScCAT1* increased at 6 h and reached the maximum level at 48 h (1.5 times that in Liucheng03–182). As previous reported, the phenomenon of smut hypha entry into the sugarcane bud meristem occurs between 6 h and 36 h after the teliospore deposition [48]. It should be also noted that *ScCAT1* expression decreased gradually after 48 h, but the expression level still maintained at a higher level than that at 0 h, and the gene expression pattern of *ScCAT1* was coincident with the activity change of catalase in this study. So we assume that *ScCAT1* may have a protective effect on smut penetration in sugarcane.

Q-PCR analysis of the expression of *ScCAT1* in response to hydrogen peroxide and plant hormones showed that from 0 h to 24 h its levels increased under the stresses of 10 mM H_2_O_2_, 5 mM SA, 25 µM MeJA and 100 µM ABA (Fig. 7A). In *Panax ginseng, PgCat1* transcript accumulated during 1–12 h of 10 mM H_2_O_2_ treatment [11]. Maize *Cat1* gene transcript increased in developing embryos by the treatments of 1.5 mM SA, 50 mM H_2_O_2_, 100 µM JA and 1 mM ABA [38], [49]. In the present study, for hyper-osmotic stress, *ScCAT1* mRNA levels increased until 12 h then slightly decreased at 24 h and induced at 48 h under 250 mM NaCl treatment. *ScCAT1* transcript was also stimulated till 24 h after 25% PEG stress (Figs. 7B and C). 500 mM NaCl stress induced the expression of *Cat1* in *Avicennia marina* seedlings till 12 h then subsequently decreased [50]. In *Panax ginseng, PgCat1* transcripts accumulated till 24 h then decreased till 72 h after 100 mM NaCl treatment [11]. Plants suffering from NaCl stress not only because of increased osmolarity but also oxidative stress caused by ionic character [51]. In our study, the *ScCAT1* transcript increased 1.5 fold until 48 h under the stress of 100 µM CuCl_2._ The maximum expression was observed to be 3.0 fold at 12 h after treatment. Previous study revealed that copper toxicity caused ultra structural damage which resulting in the increasing production of ROS [46]. The *Prunus cerasifera Cat1* gene expression and enzyme activity were high for 10 days under 100 mM copper stress [52]. These results lead us to conclude that *ScCAT1* may be a positive responsive component of abiotic stresses in sugarcane.

*N. benthamiana* has been widely employed in functional characterization of the target genes by over-expression [30]. Cell death presented at the infected site is the most efficient strategy to restrict pathogen growth and development [30]. The induction of R gene expression, ion fluxes, stimulation of ROS and defense-related hormones, are the common response of cell death [53], [54]. Here, DAB staining showed deep brown in the presence of H_2_O_2_ in *N. benthamiana* leaves after 48 h infiltration and resulted in an increase of electrolyte leakage (Figs. 8A and C). Trypan blue staining exhibited a darker color post 5 d injection than that in mock (Fig. 8B). Previous studies have shown that there is a close relationship between HR and H_2_O_2_ accumulation [55]. It can be deduced from this study that H_2_O_2_ accumulation by transient over-expression of *ScCAT1* may confer the HR cell death in sugarcane.

In conclusion, after inoculation with *S. scitamineum,* sugarcane catalase was found to significantly increase in the resistant variety and maintain at much higher level than that of the susceptible one which suggested a positive correlation between the activity of the catalase and the smut resistance in sugarcane. *ScCAT1* was isolated from sugarcane buds and the recombinant protein resulted in a better growth of *E. coli* Rosetta cells under certain stresses. The expression of *ScCAT1* was up-regulated by smut infection and by different stresses such as plant hormones (SA, MeJA and ABA) treatments, oxidative (H_2_O_2_) stress, heavy metal (CuCl_2_) and hyper-osmotic (PEG and NaCl) stresses. ScCAT1 was located in plasma membrane and cytoplasm in cells. Histochemical assays indicated that *ScCAT1* acted positively in sugarcane immunity. From these observations, we can conclude that *ScCAT1* should be a positive responsive component of biotic and abiotic stresses in sugarcane.

## Supporting Information

Figure S1 **Construction of subcellular localization vector** ***35S*** **::** ***ScCAT1*** **::** ***GFP*** **.**
(TIF)

Figure S2 **Nucleotide acid sequences and deduced amino acid sequences of** ***ScCAT1*** **obtained by RT-PCR.** The deduced amino acid sequences were shown in one-letter code under the cDNA sequences. The underlines showed the catalase active site signature (FARERIPERVVHARGAS) and the heme-ligand signature (RVFAYADTQ) of ScCAT1.
(TIF)

Figure S3 **The enzyme digestion to identify the insert-integrated subcellular localization expression vector** ***35S*** **::** ***ScCAT1*** **::** ***GFP*** **.** 1, 15,000+2,000 bp DNA marker; 2, *35S*::*GFP*/*Xba* I; 3, *ScCAT1* ORF PCR product; 4, *35S*::*ScCAT1*::*GFP*/*Xba* I; 5, *35S*::*ScCAT1*::*GFP*/*Xba* I+*Spe* I; 6, 100 bp ladder DNA marker.
(TIF)

Figure S4 **The enzyme digesting identification of insert-integrated prokaryotic expression vector pET 32a-** ***ScCAT1*** **(A) and corresponding protein expressions in** ***Escherichia coli*** **Rosetta strains (B). (A)** 1, 100 bp ladder DNA marker; 2, pET 32a/*Eco*R I; 3, *ScCAT1* ORF PCR product; 4, pET 32a-*ScCAT1*/*Eco*R I; 5, pET 32a-*ScCAT1*/*Eco*R I+*Xho* I; 6, 15,000+2,000 bp DNA Marker. **(B)** 1, Protein marker; 2, blank without induction; 3, blank induction for 8 h; 4, control without induction; 5, control induction for 8 h; 6, pET 32a-*ScCAT1* without induction; 7 and 8, pET 32a-*ScCAT1* induction for 4 h and 8 h, respectively. The induced protein was shown by arrow.
(TIF)
