# Supplementary figures and images for: Isolation of a Novel Peroxisomal Catalase Gene from Sugarcane, Which Is Responsive to Biotic and Abiotic Stresses
Source: PLoS One. 2014 Jan 2;9(1):e84426. doi: 10.1371/journal.pone.0084426 (PMC3879312; doi:10.1371/journal.pone.0084426)

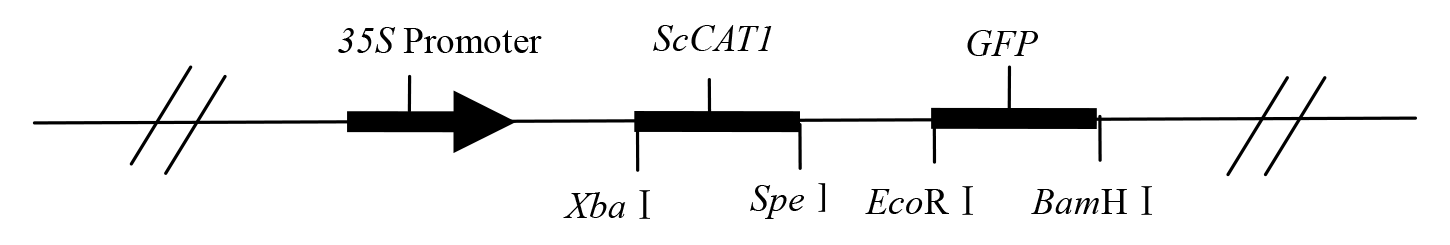

Supplement: Figure S1 — Construction of subcellular localization vector 35S :: ScCAT1 :: GFP . (TIF) [file pone.0084426.s001.tif]

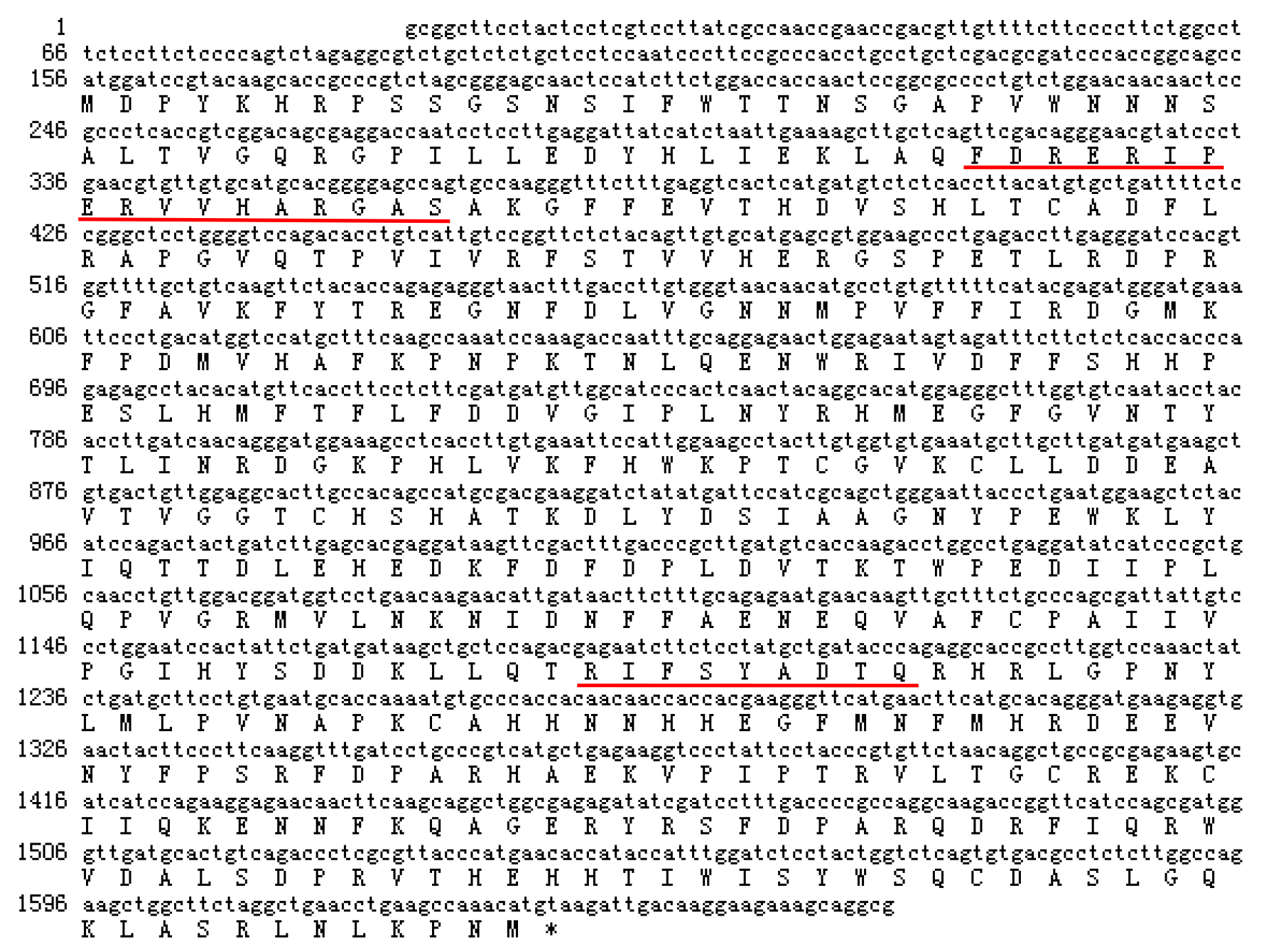

Supplement: Figure S2 — Nucleotide acid sequences and deduced amino acid sequences of ScCAT1 obtained by RT-PCR. The deduced amino acid sequences were shown in one-letter code under the cDNA sequences. The underlines showed the catalase active site signature (FARERIPERVVHARGAS) and the heme-ligand signature (RVFAYADTQ) of ScCAT1. (TIF) [file pone.0084426.s002.tif]

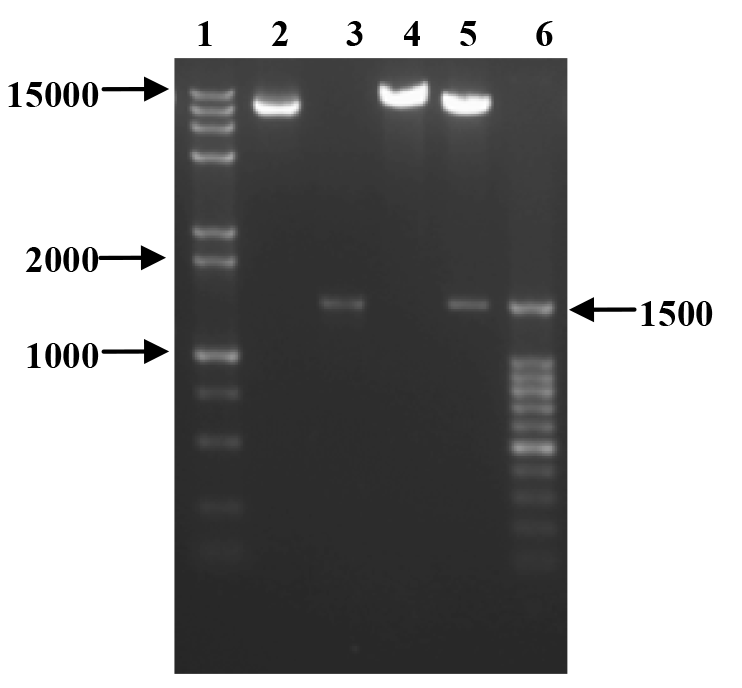

Supplement: Figure S3 — The enzyme digestion to identify the insert-integrated subcellular localization expression vector 35S :: ScCAT1 :: GFP . 1, 15,000+2,000 bp DNA marker; 2, 35S::GFP/Xba I; 3, ScCAT1 ORF PCR product; 4, 35S::ScCAT1::GFP/Xba I; 5, 35S::ScCAT1::GFP/Xba I+Spe I; 6, 100 bp ladder DNA marker. (TIF) [file pone.0084426.s003.tif]

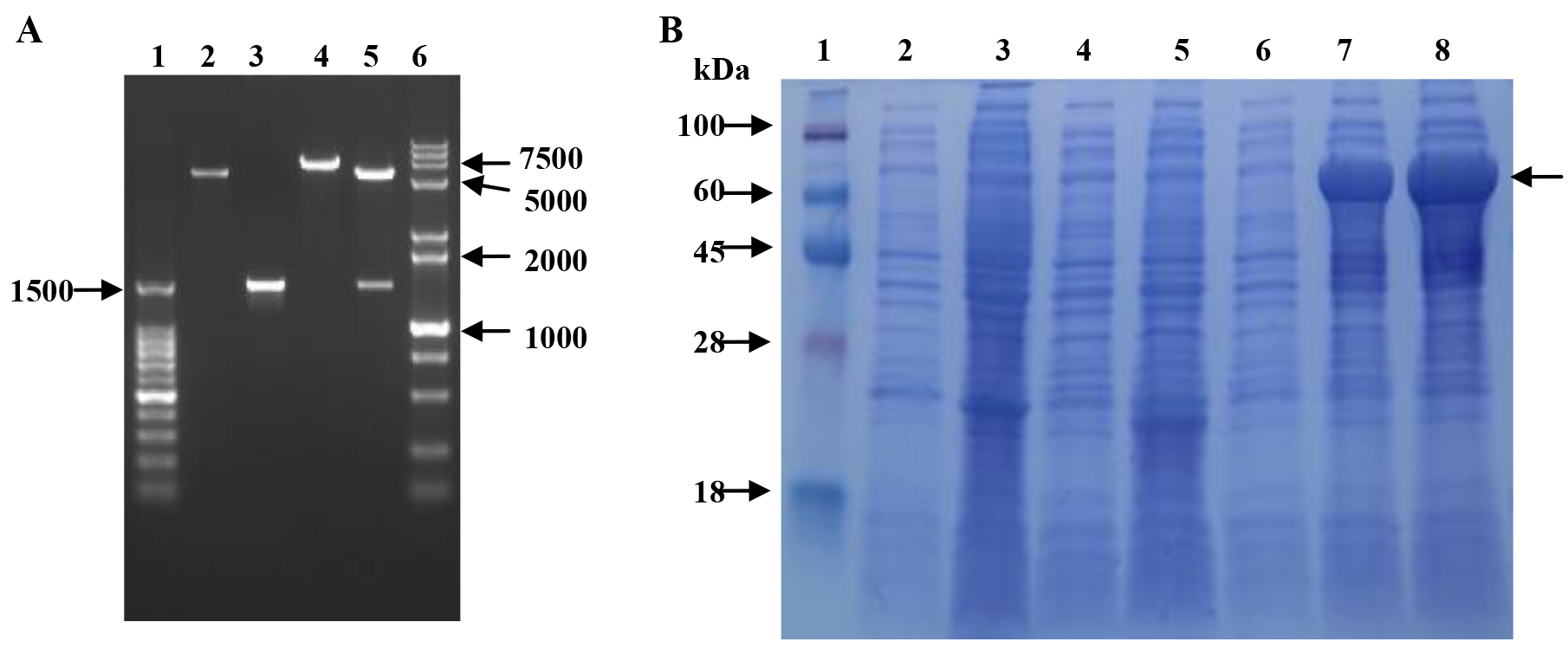

Supplement: Figure S4 — The enzyme digesting identification of insert-integrated prokaryotic expression vector pET 32a- ScCAT1 (A) and corresponding protein expressions in Escherichia coli Rosetta strains (B). (A) 1, 100 bp ladder DNA marker; 2, pET 32a/EcoR I; 3, ScCAT1 ORF PCR product; 4, pET 32a-ScCAT1/EcoR I; 5, pET 32a-ScCAT1/EcoR I+Xho I; 6, 15,000+2,000 bp DNA Marker. (B) 1, Protein marker; 2, blank without induction; 3, blank induction for 8 h; 4, control without induction; 5, control induction for 8 h; 6, pET 32a-ScCAT1 without induction; 7 and 8, pET 32a-ScCAT1 induction for 4 h and 8 h, respectively. The induced protein was shown by arrow. (TIF) [file pone.0084426.s004.tif]
